# Structural Features of the ATP-Binding Cassette (ABC) Transporter ABCA3

**DOI:** 10.3390/ijms160819631

**Published:** 2015-08-19

**Authors:** Alessandro Paolini, Antonella Baldassarre, Ilaria Del Gaudio, Andrea Masotti

**Affiliations:** Bambino Gesù Children’s Hospital-IRCCS, Gene Expression-Microarrays Laboratory, Viale di San Paolo 15, 00146 Rome, Italy; E-Mails: alepaolini86@gmail.com (A.P.); antonella.baldassarre@opbg.net (A.B.); iladile@libero.it (I.D.G.)

**Keywords:** ABC transporters, ABCA3, protein model

## Abstract

In this review we reported and discussed the structural features of the ATP-Binding Cassette (ABC) transporter ABCA3 and how the use of bioinformatics tools could help researchers to obtain a reliable structural model of this important transporter. In fact, a model of ABCA3 is still lacking and no crystallographic structures (of the transporter or of its orthologues) are available. With the advent of next generation sequencing, many disease-causing mutations have been discovered and many more will be found in the future. In the last few years, ABCA3 mutations have been reported to have important pediatric implications. Thus, clinicians need a reliable structure to locate relevant mutations of this transporter and make genotype/phenotype correlations of patients affected by ABCA3-related diseases. In conclusion, we strongly believe that the model preliminarily generated by these novel bioinformatics tools could be the starting point to obtain more refined models of the ABCA3 transporter.

## 1. Introduction

ATP-binding cassette transporters (ABC transporters) constitute one of the largest known superfamilies of proteins, that are well represented in all species, from prokaryotes to man. These proteins are classified according to the sequence and organization of their ABC domain(s) [1].

In eukaryotes, ABC transporters are expressed in plasma membrane, in intracellular compartments such as Golgi, in endosomes, in multivesicular bodies, in the endoplasmic reticulum, in peroxisomes, and mitochondria [2]. In humans, 48 members of ABC transporters have been identified so far, that have been subdivided into seven families (called ABC A–G) according to their structural features. ABC proteins are mainly involved in molecular trafficking processes, such as the transport of vitamins, lipids (*i.e.*, cholesterol, phospholipids, glycolipids, *etc*.), bile salts, steroids, toxins, drugs, and metabolities, across biological membranes [3]. Accumulating evidence supports the fact that the ABC transporter’s A-subfamily have a crucial role in human physiology, as they cause various diseases when mutated or altered [4]. Examples of ABC A-subfamily disorders include Tangier’s (ABCA1), Alzheimer’s (ABCA2/ABCA7), Stargardt’s (ABCR/ABCA4), and Harlequin Ichthyosis (ABCA12) [5].

In this mini-review we focused our interest on ABCA3 transporter for its involvement in pediatric diseases such as neonatal surfactant deficiency [6] and to collect information on the structural features of this fundamental protein. In fact, we strongly believe that increasing our knowledge about the structural features (crystallographic structure or protein bioinformatics models) of this protein would allow researchers and clinicians to face many unresolved questions [7]. The precise localization of genomic mutations already described would allow obtaining insight into the precise function of the various ABCA3 sub-domains in the export process. Moreover, a definite crystallographic structure would also allow the assessment of the interaction of ABCA3 with ATP, substrates and/or other proteins, the presence of other intra-/inter-molecular connections, or to verify/exclude the presence of other genetic or environmental factors concurring to disease pathogenesis.

## 2. Functional Features of ABC Transporters

ABC transporters (ABCs) are active transporters; that is, they require energy in the form of adenosine triphosphate (ATP) to translocate substrates across cell membranes. ABCs bind and hydrolyze ATP, producing energy that they use to transfer compounds across the membrane or to flip molecules from the inner to the outer leaflet of the membranes [2]. ABC transporters are divided into three main functional categories: importers (*i.e.*, in prokaryotes they mediate the uptake of nutrients into the cell), exporters (in eukaryotes and prokaryotes they export various types of molecules), and ABCs involved in translation and DNA repair processes. ABC importers and exporters have quite similar transport mechanisms (*i.e.*, they open and close transmembrane domains and allow the transport of the substrate) and this suggests that, also, the structures should be quite similar [8].

ABC transporters, subfamily A in particular, have a characteristic architecture, which consists mainly of four domains: two transmembrane domains (TMD1 and TMD2), spanning the membrane bilayer, and two nucleotide-binding domains (NBD1 and NBD2), located in the cytoplasm [1,4,9]. The TMD consists of six α-helices able to recognize a variety of substrates that undergoes conformational changes to transport the substrate across the membrane. At the sequence level, the superfamily of ABC transporters present highly conserved motifs within the NBDs; in contrast, the sequences and architectures of the TMDs are quite variable, reflecting the chemical diversity of the translocated substrates. The NBD is the site for ATP binding. Other additional regulatory elements (*i.e.*, binding proteins) may be associated with specific ABC transporters [10].

### 2.1. Mechanism of Transport

The mechanism of transport by importers supports the alternating-access model. Briefly, the resting state of importers is characterized by an inwardly-facing conformation, where the two NBDs are kept open by the TMDs that face outward. When the substrate enters the transporter, transmembrane domains change their conformation and ATP can bind to NBDs. These events allow the transporter to switch into an outward-facing conformation in which the TMDs have reoriented to receive the substrate from the binding protein. After hydrolysis of ATP into ADP and Pi, the NBD dimer opens, the substrate is released into the cytoplasm, and the transporter is again converted into the resting state [8,10,11,12]. For exporters, the transport cycle begins with substrate binding to an inward-facing, open NBD conformation of the ABC transporter. This is followed by ATP-dependent closure of the NBDs, which concomitantly can shift the transporter to an outward-facing conformation and exposes the substrate for release on the other side of the membrane. Here, ATP hydrolysis leads to NBDs reopening and the transporter returns to the initial conformation [10,13,14].

The ABCA3 protein belongs to the latter family and is involved in the export of phospholipids (*i.e.*, phosphatidylcholine and phosphatidylglycerol). Specifically, ABCA3 has been localized, predominantly, to the limiting membrane of lamellar bodies that are lipid-rich organelles associated with the production, storage, and secretion of pulmonary surfactant through the generation of lamellar body-like structures [15,16]. Once into lamellar bodies, these lipids interact with other surfactant proteins (*i.e.*, SP-B and SP-C) and form the surfactant. Lamellar bodies are then extruded from lung epithelial type II cells into the alveolar lumen via exocytosis [15,17,18].

ABCA3 is highly expressed in type II alveolar epithelial cells, liver, stomach, kidney, pancreas, and brain. A growing body of evidence during the last decade has suggested that the dysfunction of cellular lipid transport and homeostasis is often associated with human diseases [19]. Although ABCA3 is widely expressed, in our review we have decided to focus and discuss only the pulmonary phenotypes.

### 2.2. In Vitro and in Vivo Models to Study the Functional Role of ABCA3

In humans, mutation of the *ABCA3* gene is the most common cause of surfactant deficiency [17], dysfunction [20], and chronic interstitial lung diseases [21]. In 185 infants and children, more than 180 distinct mutations of *ABCA3* including multiple-missense, splice-site, and frameshift have been found associated with severe pediatric neonatal respiratory disorders and other interstitial lung diseases [17,22,23,24].

To study the functional role of these mutations, several *in vitro* and *in vivo* models have been reported in the literature. One of these *in vitro* studies demonstrated that the p.N568D mutation in ABCA3 impairs choline-phospholipids uptake into intracellular vesicles in lung adenocarcinoma A549 cells [25] and primary AT2 cells [26]. Moreover, in WT cells’ ultrastructural images confirmed the regular formation of lamellar bodies, whereas in the p.N568D mutant the formation of these multilamellar vesicles has not been observed.

ABCA3 is also critical for lamellar body biogenesis *in vivo* [27]. Knock-out and engineered mice models have greatly contributed to the understanding of the role of ABCA3 in surfactant biogenesis and lamellar body formation [28,29]. Mice with a mutated or disrupted *Abca3* gene display a phenotype resembling that observed in humans with a similar genotype [28,30,31]. Homozygous *Abca3*^−/−^ knock-out mice died soon after birth [28] as a result of the inability to secrete pulmonary surfactant into the alveolar space and probably due to the aberrant processing of surfactant proteins, as well. On the contrary, most of *Abca3* wild type and heterozygous *Abca3*^+/−^ animals survived [27]. Alveolar type 2 cells from *Abca3*^−/−^ embryos contained no lamellar bodies, and expression of mature SP-B protein was disrupted when compared with the normal lung surfactant system of wild type embryos. Similarly, Ban *et al*. engineered mice by disrupting the first nucleotide-binding domain of Abca3 protein and observed that lung tissue showed an impaired differentiation of pulmonary epithelial cells that contained small lamellar body-like organelles with dense peripheral inclusions [31,32]. Finally, the normal expression of Abca3 is required to maintain lipid homeostasis and surfactant function also in adult respiratory epithelial cells, whereas Abca3 deficiency resulted in cell injury and lung remodeling [33].

## 3. Structural Features of ABCA3

Adenosine triphosphate (ATP)-binding cassette A3 transporter (ABCA3) is a member of the evolutionarily highly conserved family of ABC transporters. Twelve human ABC transporter genes belonging to the A-subfamily (*ABCA1-ABCA10*, *ABCA12*, and *ABCA13*) and two pseudogenes (*ABCA11P* and *ABCA17P*) are known so far [34,35]. The *ABCA3* gene (more than 80 kb length) maps to human chromosome 16 (16p13.3) and consists of 33 exons (30 of which are coding) encoding a 1704 amino acid (~150 kDa) protein.

**Figure 1:**
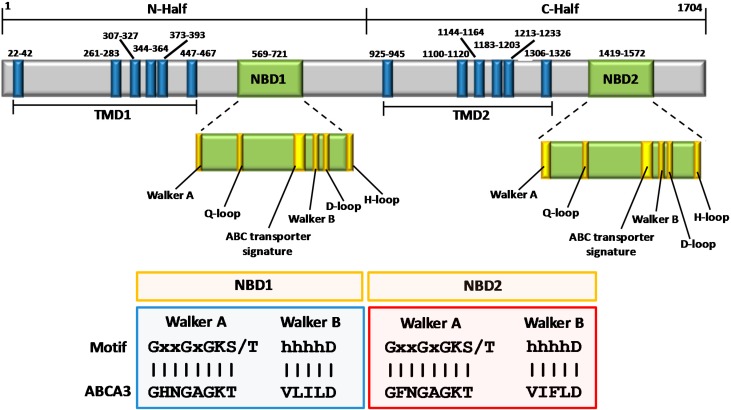
Schematic representation of ABCA3 protein with domains indicated in blue (α-helices) and green (NBDs). The N- and C-half have been indicated and the motif sequences of Walker A and B for NBD1 and NBD2 have been reported and compared to that of ABCA3. ABCA3: adenosine triphosphate (ATP)-binding cassette A3 transporter; NBD: nucleotide-binding domains; TMD: transmembrane domains.

The ABCA3 protein (NP_001080.2) has a molecular weight of 191.39 kDa and is organized into two tandem functional units (N-half and C-half), consisting in a TMD (α-helix motif) and a NBD for each half (Figure 1) [36,37].

Both N- and C-halves of ABCA3 have been predicted to contain a TMD formed by six hydrophobic membrane-spanning α-helices connected by a long loop (156–220 aa) connecting the first and the second α-helix, and by short hydrophilic loops (8–53 aa length) linking the remaining helices of the TMS. The six transmembrane helices in N- and C-halves have an average length of 21 amino acids (Figure 1). The extracellular domain (ECD) loops and the TMDs constitute the main substrate binding site that allows the trafficking of lipid molecules.

As in other ABCA proteins, ABCA3 presents a short (~22 aa) N-terminal protein segment of positively-charged residues, which is followed by the first α-helix of the transmembrane motif: a domain rich in hydrophobic amino acids such as valine and leucine. The extracellular domains (ECDs) between the first α-helix (N- and C-half) and the rest of the TMD domain protrude in the extracellular compartment. The length of ECD1 and ECD2 is 220 and 156 amino acids in N-half and C-half, respectively (Figure 1). However, in ABCA3 protein the function of these two extracellular loops is still unknown. Further studies are needed to explore the possibility that other molecules may interact with ABCA3 such as in the case of apolipoprotein (apo) A-I to ABCA1 [38]. Furthermore, it should not be excluded that inter-helical loops of ABCA3 might play an important structural and functional role, in analogy to what observed for MsbA and BtuC in prokaryotes [39,40]. Most of the members of ABC transporters have a similar domain organization except for the number of transmembrane domains that may vary (*i.e.*, ABCB2, ABCC1, *etc*.) or the different length of the loops that connect them. The distance between the last α-helix and the NBD (for the N- and C-half) is of 103 and 94 aa, respectively (Figure 1).

**Figure 2:**
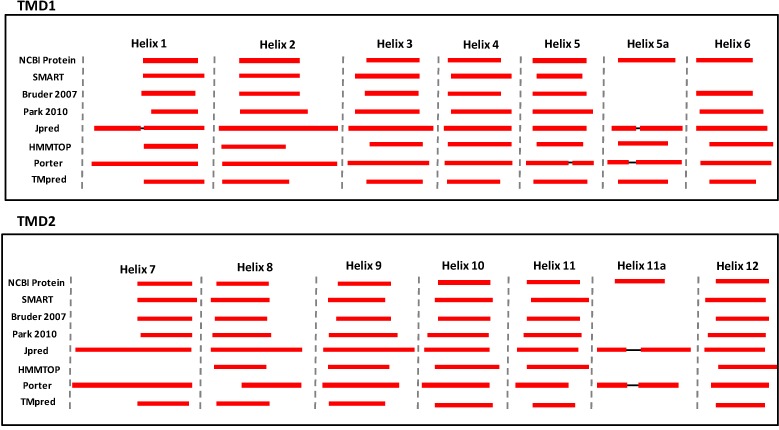
The α-helices of the TMD1 and TMD2 predicted by various bioinformatics software are indicated (red blocks).

Of note, the two most common graphical representations proposed to depict the structure of ABCA3 have been obtained in different ways [36,37]. The procedures followed to determine these models determined the small differences in the numbering used to identify the various domains (α-helices and NBDs). Moreover, apart from the two models currently used to depict the structure of ABCA3, other prediction algorithms such as SMART [41], JPred [42], HMMTOP [43], Porter [44], and TMpred [45] may predict slightly different coordinates (Figure 2 and Supplementary Figure S1).

Surprisingly, NCBI Protein graphics, JPred and Porter report the presence of an additional region where other α-helix could be present in both N- and C-half (5a and 11a in Figure 2), making a clear depiction of ABCA3 structure very challenging. However, these regions have been suggested to have a low prediction score and should not be considered real α-helix motifs [4]. In fact, owing to the lack of a crystal structure determination of this protein, an exact localization of these domains and their functional role is not yet possible.

**Figure 3:**
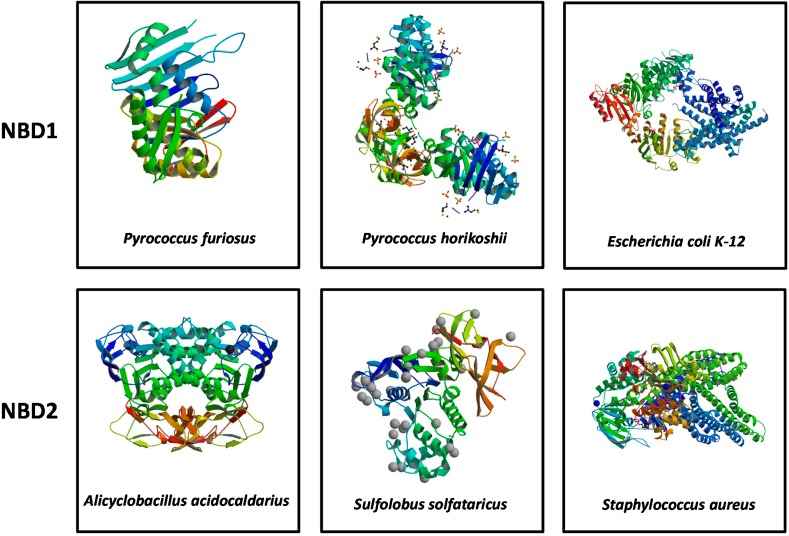
The Crystal structure of NBD1 and NBD2 in various organisms: *Pyrococcus furiosus*, *Pyrococcus horikoshii*, *Escherichia coli K-12*, *Alicyclobacillus acidocaldarius*, *Sulfolobus solfataricus*, and *Staphylococcus aureus*.

The ABCA3 nucleotide binding domains (NBD1 and NBD2) consist of 153 and 154 amino acids, respectively and are generally conserved in other ABC proteins of many species (Figure 3) [46].

The NBD includes seven motifs: A-loop, Walker A motif, Q-loop, ABC signature, Walker B, D-loop, and H-loop (Figure 1) [13]. The motif of Walker B region is hhhhD (h is hydrophobic amino acids) which in ABCA3 is VLILD for NBD1 and VIFLD for NBD2.

In ABCA3, the A-loop consists of hydrophobic residues, where the Phe639 and the Tyr1390 might be involved in stacking interactions with the adenine ring of ATP [47]. The Walker A motif is characterized by the motif GxxGxGKS/T (x is any amino acid) that in ABCA3 is GHNGAGKT for NBD1 and GFNGAGKT for NBD2. The Q-loop is another important region for the binding of ATP in the NBDs. The amino acids 613 and 1463 seem to have an essential role in the functionality of the ABC protein being the site of major conformational change during the closure of NBDs [48]. The function of the D-loop in ABCA3 is not completely clear although many studies suggest that amino acids in position 696 and 1546 could be involved in the cross-talk between the two NDBs [49] and in the stabilization of hydrogen bonds of the Walker A loop with the γ-phosphate of ATP [50]. Finally, histidine residues of the ABCA3 H-loop in position 723 and 1574 seem to be essential in shuttling protons between reactants during the catalysis of ATP hydrolysis [51].

Homology modelling studies suggest that NBDs of ABCA3 have a structural organization similar to other ABC transporters [52,53], although the 3D crystal structure of this ABC transporter is not available. We performed preliminary secondary structure comparative analyses on the ABCA3 protein and we found that NBD1 and NBD2 respectively have 35% and 38% similarity with the correspondent NBDs of *Thermotoga maritima*, and a similarity of 28% and 23% respectively with NBDs of human ABCB10 [54] (Supplementary Figure S2). Moreover, NBD1 shares 39%, 36% and 28% similarity with the *Pyrococcus furiosus* Rad 50 ABC-ATPase, the *Pyrococcus horikoshii* multiple sugar binding transport ATP-binding protein and the *Escherichia coli K-12* MetNI methionine ABC transporter, respectively (Figure 3) [49,55]. Similarly, NBD2 shares 31%, 26% and 22% similarity with the *Alicyclobacillus acidocaldarius* ATPase subunit CysA of the putative sulfate ATP-binding cassette, the *Sulfolobus solfataricus* GlcV and the *Staphylococcus aureus* Multidrug ABC transporter SAV1866, respectively (Figure 3) [56,57,58].

**Figure 4:**
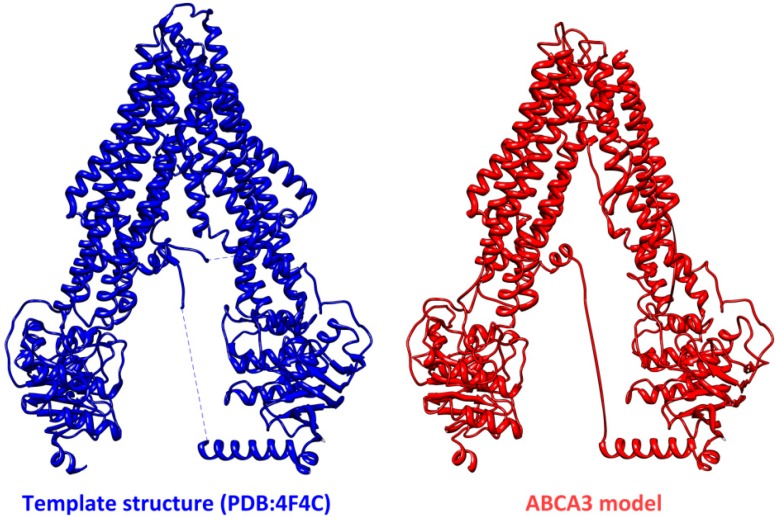
The template structure and the ABCA3 model obtained by Phyre2.

To gain further insights into the structure of ABCA3, we entered the full-length sequence of ABCA3 into the web interface of Phyre2 [59] and we obtained a preliminary 3D structural model only for a portion of ABCA3 (from Asp253 to Lys1604). The software returned the highest scoring template used to model the ABCA3 sequence, which consisted in the ATP-binding cassette transporter protein *P*-gp, a *P*-glycoprotein from *Caenorhabditis elegans* (PDB:4F4C) [60]. Up to 1061 residues (62% of ABCA3 sequence) have been modelled with 100.0% confidence and the resulting model has been reported in Figure 4 together with the template structure.

Without pretending to be exhaustive, the preliminary model that we presented here is only an example of the output that can be obtained. The novel computational tools that we used in this preliminary work will be further exploited by our group to obtain a more realistic model of this transporter. In fact, having a reliable 3D model or a crystal structure of this protein is of paramount importance to help determine genotype-phenotype correlations for individuals with ABCA3 mutations.

**Figure 5:**
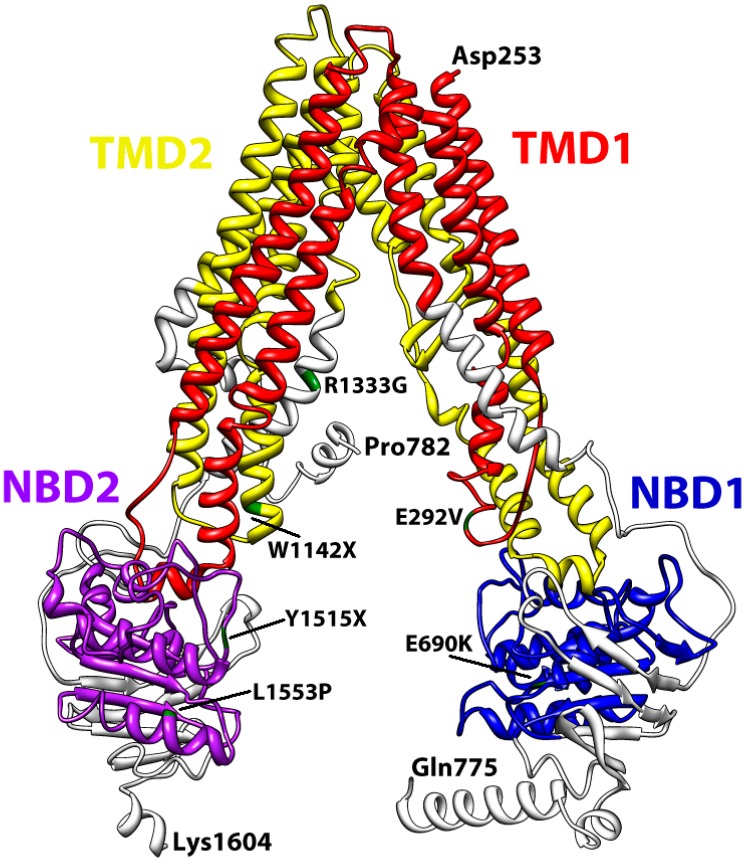
The color-coded 3D model of ABCA3 and the localization of some of the most frequent mutations reported in the literature [61].

We report here some examples of the significance of this model by localizing six mutations reported by Wambach [24] within the protein structural model. In particular, we focused on the following mutations: p.E292V, p.E690K, p.R1333G, p.W1142X, p.Y1515X, and p.L1553P. By using our model (Figure 5), we were able to localize these mutations and, for some of them, to suggest their possible role in ABCA3 functioning. The p.E292V mutation, one of the most frequently observed in humans [61], is localized in a loop very close to the terminal part of the transporter channel and we may hypothesize that this amino acid is involved in ATP or substrate binding. p.E690K reside in the NBD1, whereas p.Y1515X and p.L1553P are localized in the NBD2. Intuitively, these mutations should contribute to impair the binding of ATP molecule. More difficult is to hypothesize a role for p.W1142X and p.R1333G both residing in α-helices of the transmembrane domain 2 (TMD2).

## 4. Conclusions

Although considerable progress has been made in understanding the role of ABCA3 in pulmonary surfactant deficiencies, phospholipid homeostasis, and respiratory distress syndromes, many experimental issues have still to be solved. One of the most important open questions is the lack of a 3D crystallographic structure of the ABCA3 transporter, which would help to obtain information on conformational changes occurring during substrate transport and/or ATP hydrolysis, in physiological and pathological conditions. Moreover, detailed modeling studies of ABCA3 might help to shed light on structure-function relationships and to assess the role of disease-associated mutations. In conclusion, we believe that, in the next few years, we will be committed to study the ABCA3 functional role in disease through next-generation sequencing and structural modeling (or crystallography) techniques in order to allow clinicians and researchers to devise novel targeted therapeutic strategies and individualized therapies.

## Supplementary Materials

Supplementary materials can be found at http://www.mdpi.com/1422-0067/16/08/19631/s1.

## References

[1] Pohl A., Devaux P.F., Herrmann A. (2005). Function of prokaryotic and eukaryotic ABC proteins in lipid transport. Biochim. Biophys. Acta.

[2] Dean M., Annilo T. (2005). Evolution of the ATP-binding cassette (ABC) transporter superfamily in vertebrates. Annu. Rev. Genom. Hum. Genet..

[3] Molday R.S., Zhong M., Quazi F. (2009). The role of the photoreceptor ABC transporter ABCA4 in lipid transport and Stargardt macular degeneration. Biochim. Biophys. Acta.

[4] Peelman F., Labeur C., Vanloo B., Roosbeek S., Devaud C., Duverger N., Denefle P., Rosier M., Vandekerckhove J., Rosseneu M. (2003). Characterization of the ABCA transporter subfamily: Identification of prokaryotic and eukaryotic members, phylogeny and topology. J. Mol. Biol..

[5] Tarling E.J., de Aguiar Vallim T.Q., Edwards P.A. (2013). Role of ABC transporters in lipid transport and human disease. Trends Endocrinol. Metab..

[6] Albrecht C., Viturro E. (2007). The ABCA subfamily—Gene and protein structures, functions and associated hereditary diseases. Pflug. Arch..

[7] Peca D., Boldrini R., Johannson J., Shieh J.T., Citti A., Petrini S., Salerno T., Cazzato S., Testa R., Messina F. (2015). Clinical and ultrastructural spectrum of diffuse lung disease associated with surfactant protein C mutations. Eur. J. Hum. Genet..

[8] Davidson A.L., Dassa E., Orelle C., Chen J. (2008). Structure, function, and evolution of bacterial ATP-binding cassette systems. Microbiol. Mol. Biol. Rev..

[9] Holland I.B., Blight M.A. (1999). ABC-ATPases, adaptable energy generators fuelling transmembrane movement of a variety of molecules in organisms from bacteria to humans. J. Mol. Biol..

[10] Rees D.C., Johnson E., Lewinson O. (2009). ABC transporters: The power to change. Nat. Rev. Mol. Cell Biol..

[11] Higgins C.F., Linton K.J. (2004). The ATP switch model for ABC transporters. Nat. Struct. Mol. Biol..

[12] Oldham M.L., Davidson A.L., Chen J. (2008). Structural insights into ABC transporter mechanism. Curr. Opin. Struct. Biol..

[13] Linton K.J., Higgins C.F. (2007). Structure and function of ABC transporters: The ATP switch provides flexible control. Pflug. Arch..

[14] Ter Beek J., Guskov A., Slotboom D.J. (2014). Structural diversity of ABC transporters. J. Gen. Physiol..

[15] Nagata K., Yamamoto A., Ban N., Tanaka A.R., Matsuo M., Kioka N., Inagaki N., Ueda K. (2004). Human ABCA3, a product of a responsible gene for ABCA3 for fatal surfactant deficiency in newborns, exhibits unique ATP hydrolysis activity and generates intracellular multilamellar vesicles. Biochem. Biophys. Res. Commun..

[16] Yamano G., Funahashi H., Kawanami O., Zhao L.X., Ban N., Uchida Y., Morohoshi T., Ogawa J., Shioda S., Inagaki N. (2001). ABCA3 is a lamellar body membrane protein in human lung alveolar type II cells. FEBS Lett..

[17] Shulenin S., Nogee L.M., Annilo T., Wert S.E., Whitsett J.A., Dean M. (2004). *ABCA3* gene mutations in newborns with fatal surfactant deficiency. N. Engl. J. Med..

[18] Mulugeta S., Gray J.M., Notarfrancesco K.L., Gonzales L.W., Koval M., Feinstein S.I., Ballard P.L., Fisher A.B., Shuman H. (2002). Identification of LBM180, a lamellar body limiting membrane protein of alveolar type II cells, as the ABC transporter protein ABCA3. J. Biol. Chem..

[19] Stahlman M.T., Besnard V., Wert S.E., Weaver T.E., Dingle S., Xu Y., von Zychlin K., Olson S.J., Whitsett J.A. (2007). Expression of ABCA3 in developing lung and other tissues. J. Histochem. Cytochem..

[20] Wert S.E., Whitsett J.A., Nogee L.M. (2009). Genetic disorders of surfactant dysfunction. Pediatr. Dev. Pathol..

[21] Doan M.L., Guillerman R.P., Dishop M.K., Nogee L.M., Langston C., Mallory G.B., Sockrider M.M., Fan L.L. (2008). Clinical, radiological and pathological features of ABCA3 mutations in children. Thorax.

[22] Garmany T.H., Moxley M.A., White F.V., Dean M., Hull W.M., Whitsett J.A., Nogee L.M., Hamvas A. (2006). Surfactant composition and function in patients with ABCA3 mutations. Pediatr. Res..

[23] Whitsett J.A., Wert S.E., Weaver T.E. (2010). Alveolar surfactant homeostasis and the pathogenesis of pulmonary disease. Annu. Rev. Med..

[24] Wambach J.A., Casey A.M., Fishman M.P., Wegner D.J., Wert S.E., Cole F.S., Hamvas A., Nogee L.M. (2014). Genotype-phenotype correlations for infants and children with ABCA3 deficiency. Am. J. Respir. Crit. Care Med..

[25] Matsumura Y., Sakai H., Sasaki M., Ban N., Inagaki N. (2007). ABCA3-mediated choline-phospholipids uptake into intracellular vesicles in A549 cells. FEBS Lett..

[26] Cheong N., Madesh M., Gonzales L.W., Zhao M., Yu K., Ballard P.L., Shuman H. (2006). Functional and trafficking defects in ATP binding cassette A3 mutants associated with respiratory distress syndrome. J. Biol. Chem..

[27] Cheong N., Zhang H., Madesh M., Zhao M., Yu K., Dodia C., Fisher A.B., Savani R.C., Shuman H. (2007). ABCA3 is critical for lamellar body biogenesis *in vivo*. J. Biol. Chem..

[28] Fitzgerald M.L., Xavier R., Haley K.J., Welti R., Goss J.L., Brown C.E., Zhuang D.Z., Bell S.A., Lu N., McKee M. (2007). ABCA3 inactivation in mice causes respiratory failure, loss of pulmonary surfactant, and depletion of lung phosphatidylglycerol. J. Lipid Res..

[29] Herber-Jonat S., Mittal R., Huppmann M., Hammel M., Liebisch G., Yildirim A.O., Eickelberg O., Schmitz G., de Angelis M.H., Flemmer A.W. (2013). Abca3 haploinsufficiency is a risk factor for lung injury induced by hyperoxia or mechanical ventilation in a murine model. Pediatr. Res..

[30] Hammel M., Michel G., Hoefer C., Klaften M., Muller-Hocker J., de Angelis M.H., Holzinger A. (2007). Targeted inactivation of the murine *Abca3* gene leads to respiratory failure in newborns with defective lamellar bodies. Biochem. Biophys. Res. Commun..

[31] Ban N., Matsumura Y., Sakai H., Takanezawa Y., Sasaki M., Arai H., Inagaki N. (2007). ABCA3 as a lipid transporter in pulmonary surfactant biogenesis. J. Biol. Chem..

[32] Tryka A.F., Wert S.E., Mazursky J.E., Arrington R.W., Nogee L.M. (2000). Absence of lamellar bodies with accumulation of dense bodies characterizes a novel form of congenital surfactant defect. Pediatr. Dev. Pathol..

[33] Besnard V., Matsuzaki Y., Clark J., Xu Y., Wert S.E., Ikegami M., Stahlman M.T., Weaver T.E., Hunt A.N., Postle A.D. (2010). Conditional deletion of *Abca3* in alveolar type II cells alters surfactant homeostasis in newborn and adult mice. Am. J. Physiol. Lung Cell. Mol. Physiol..

[34] Dean M. (2005). The genetics of ATP-binding cassette transporters. Methods Enzymol..

[35] Piehler A.P., Wenzel J.J., Olstad O.K., Haug K.B., Kierulf P., Kaminski W.E. (2006). The human ortholog of the rodent testis-specific ABC transporter Abca17 is a ubiquitously expressed pseudogene (*ABCA17P*) and shares a common 5ʹ end with *ABCA3*. BMC Mol. Biol..

[36] Bruder E., Hofmeister J., Aslanidis C., Hammer J., Bubendorf L., Schmitz G., Rufle A., Buhrer C. (2007). Ultrastructural and molecular analysis in fatal neonatal interstitial pneumonia caused by a novel ABCA3 mutation. Mod. Pathol..

[37] Park S.K., Amos L., Rao A., Quasney M.W., Matsumura Y., Inagaki N., Dahmer M.K. (2010). Identification and characterization of a novel ABCA3 mutation. Physiol. Genom..

[38] Vedhachalam C., Duong P.T., Nickel M., Nguyen D., Dhanasekaran P., Saito H., Rothblat G.H., Lund-Katz S., Phillips M.C. (2007). Mechanism of ATP-binding cassette transporter A_1_-mediated cellular lipid efflux to apolipoprotein A-I and formation of high density lipoprotein particles. J. Biol. Chem..

[39] Chang G., Roth C.B. (2001). Structure of MsbA from *E. coli*: A homolog of the multidrug resistance ATP binding cassette (ABC) transporters. Science.

[40] Locher K.P., Lee A.T., Rees D.C. (2002). The *E. coli* BtuCD structure: A framework for ABC transporter architecture and mechanism. Science.

[41] Letunic I., Doerks T., Bork P. (2015). SMART: Recent updates, new developments and status in 2015. Nucleic Acids Res..

[42] Drozdetskiy A., Cole C., Procter J., Barton G.J. (2015). JPred4: A protein secondary structure prediction server. Nucleic Acids Res..

[43] Tusnady G.E., Simon I. (2001). The HMMTOP transmembrane topology prediction server. Bioinformatics.

[44] Pollastri G., McLysaght A. (2005). Porter: A new, accurate server for protein secondary structure prediction. Bioinformatics.

[45] Hofmann K., Stoffel W. (1993). Tmbase-A database of membrane spanning protein segments. Biol. Chem. Hoppe-Seyler.

[46] Higgins C.F. (1992). ABC transporters: From microorganisms to man. Annu. Rev. Cell Biol..

[47] Kim I.W., Peng X.H., Sauna Z.E., FitzGerald P.C., Xia D., Muller M., Nandigama K., Ambudkar S.V. (2006). The conserved Tyrosine residues 401 and 1044 in ATP sites of human *P*-glycoprotein are critical for ATP binding and hydrolysis: Evidence for a conserved subdomain, the A-loop in the ATP-binding cassette. Biochemistry.

[48] Linton K.J. (2007). Structure and function of ABC transporters. Physiology.

[49] Hopfner K.P., Karcher A., Shin D.S., Craig L., Arthur L.M., Carney J.P., Tainer J.A. (2000). Structural biology of Rad50 ATPase: ATP-driven conformational control in DNA double-strand break repair and the ABC-ATPase superfamily. Cell.

[50] De la Rosa M.B., Nelson S.W. (2011). An interaction between the Walker A and D-loop motifs is critical to ATP hydrolysis and cooperativity in bacteriophage T4 Rad50. J. Biol. Chem..

[51] Zhou Y., Ojeda-May P., Pu J. (2013). H-loop histidine catalyzes ATP hydrolysis in the *E. coli* ABC-transporter HlyB. Phys. Chem. Chem. Phys..

[52] Kurashima-Ito K., Ikeya T., Senbongi H., Tochio H., Mikawa T., Shibata T., Ito Y. (2006). Heteronuclear multidimensional NMR and homology modelling studies of the C-terminal nucleotide-binding domain of the human mitochondrial ABC transporter ABCB6. J. Biomol. NMR.

[53] Ecker G.F., Stockner T., Chiba P. (2008). Computational models for prediction of interactions with ABC-transporters. Drug Discov. Today.

[54] Shintre C.A., Pike A.C.W., Li Q., Kim J.I., Barr A.J., Goubin S., Shrestha L., Yang J., Berridge G., Ross J. (2013). Structures of ABCB10, a human ATP-binding cassette transporter in apo-and nucleotide-bound states. Proc. Natl. Acad. Sci. USA.

[55] Johnson E., Nguyen P.T., Yeates T.O., Rees D.C. (2012). Inward facing conformations of the MetNI methionine ABC transporter: Implications for the mechanism of transinhibition. Protein Sci..

[56] Scheffel F., Demmer U., Warkentin E., Hulsmann A., Schneider E., Ermler U. (2005). Structure of the ATPase subunit CysA of the putative sulfate ATP-binding cassette (ABC) transporter from *Alicyclobacillus acidocaldarius*. FEBS Lett..

[57] Verdon G., Albers S.V., van Oosterwijk N., Dijkstra B.W., Driessen A.J., Thunnissen A.M. (2003). Formation of the productive ATP-Mg^2+^-bound dimer of GlcV, an ABC-ATPase from *Sulfolobus solfataricus*. J. Mol. Biol..

[58] Dawson R.J., Locher K.P. (2006). Structure of a bacterial multidrug ABC transporter. Nature.

[59] Kelley L.A., Mezulis S., Yates C.M., Wass M.N., Sternberg M.J. (2015). The Phyre2 web portal for protein modeling, prediction and analysis. Nat. Protoc..

[60] Jin M.S., Oldham M.L., Zhang Q., Chen J. (2012). Crystal structure of the multidrug transporter *P*-glycoprotein from *Caenorhabditis elegans*. Nature.

[61] Bullard J.E., Wert S.E., Whitsett J.A., Dean M., Nogee L.M. (2005). ABCA3 mutations associated with pediatric interstitial lung disease. Am. J. Respir. Crit. Care Med..

