# Supplementary material for: Structural Features of the ATP-Binding Cassette (ABC) Transporter ABCA3
Source: Int J Mol Sci. 2015 Aug 19;16(8):19631–44. doi: 10.3390/ijms160819631 (PMC4581316; doi:10.3390/ijms160819631)
Supplement: Supplementary file 1 [file ijms-16-19631-s001.pdf]

Supplementary Information

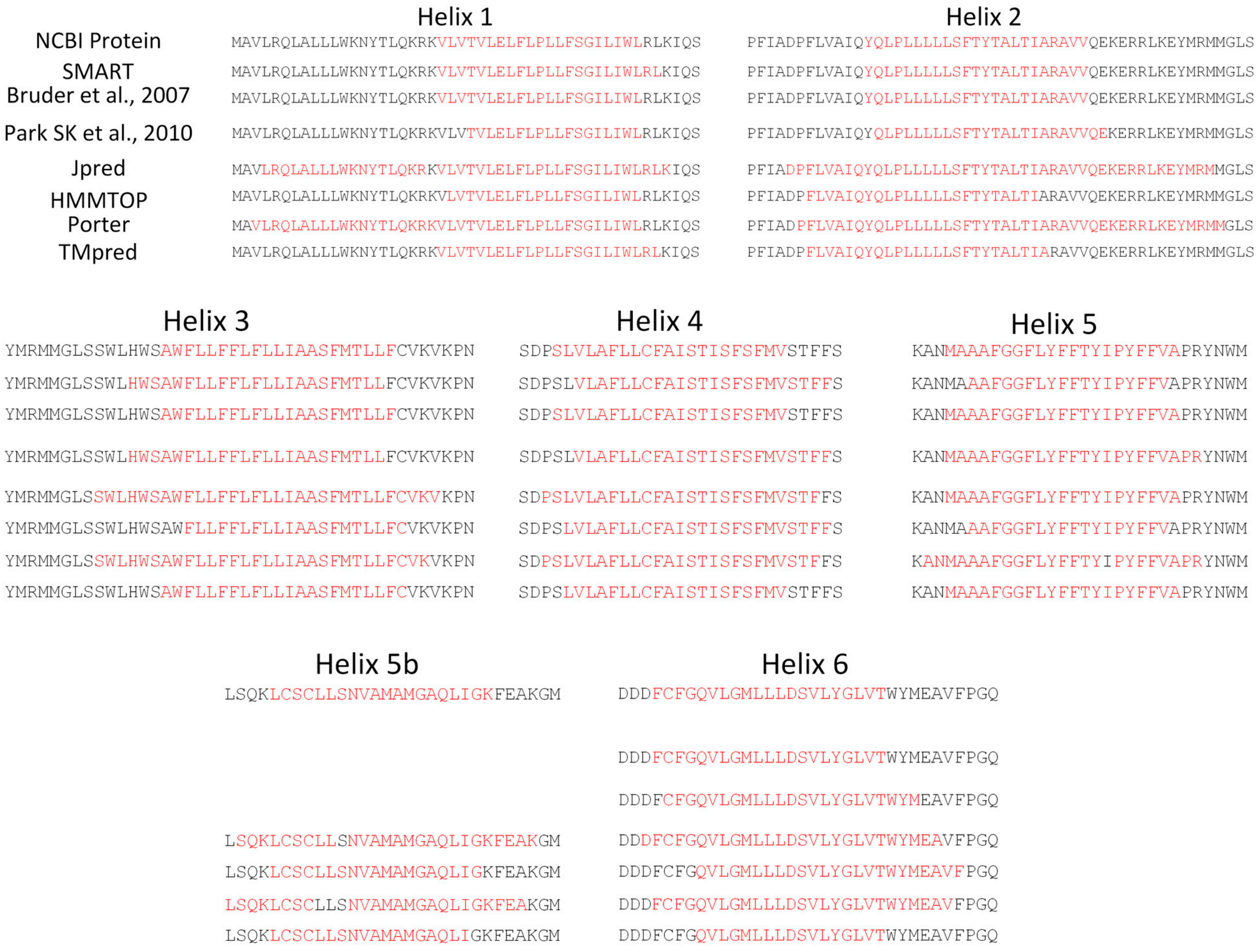

Figure S1. *Cont.*

Helix 7

```
GLALHCQQFWAMFLKKAAYSWREWKMVAAQVLVPLTCVTLALLAINYSSE
GLALHCQQFWAMFLKKAAYSWREWKMVAAQVLVPLTCVTLALLAINYSSE
GLALHCQQFWAMFLKKAAYSWREWKMVAAQVLVPLTCVTLALLAINYSSE
GLALHCQQFWAMFLKKAAYSWREWKMVAAQVLVPLTCVTLALLAINYSSE
GLALHCQQFWAMFLKKAAYSWREWKMVAAQVLVPLTCVTLALLAINYSSE

GLALHCQQFWAMFLKKAAYSWREWKMVAAQVLVPLTCVTLALLAINYSSE
GLALHCQQFWAMFLKKAAYSWREWKMVAAQVLVPLTCVTLALLAINYSSE
```

Helix 8

```
GFDIALNLLFAMAFLASTFSILAVSERAVQAKHVQFVSGV
GFDIALNLLFAMAFLASTFSILAVSERAVQAKHVQFVSGV
GFDIALNLLFAMAFLASTFSILAVSERAVQAKHVQFVSGV
GFDIALNLLFAMAFLASTFSILAVSERAVQAKHVQFVSGV
GFDIALNLLFAMAFLASTFSILAVSERAVQAKHVQFVSGV
GFDIALNLLFAMAFLASTFSILAVSERAVQAKHVQFVSGV
GFDIALNLLFAMAFLASTFSILAVSERAVQAKHVQFVSGV
GFDIALNLLFAMAFLASTFSILAVSERAVQAKHVQFVSGV
```

Helix 9

```
HVASFWLSALLWDLISFLIPSLLLLVVFKAFDVRAFTRDG
HVASFWLSALLWDLISFLIPSLLLLVVFKAFDVRAFTRDG
HVASFWLSALLWDLISFLIPSLLLLVVFKAFDVRAFTRDG
HVASFWLSALLWDLISFLIPSLLLLVVFKAFDVRAFTRDG
HVASFWLSALLWDLISFLIPSLLLLVVFKAFDVRAFTRDG
HVASFWLSALLWDLISFLIPSLLLLVVFKAFDVRAFTRDG
HVASFWLSALLWDLISFLIPSLLLLVVFKAFDVRAFTRDG
HVASFWLSALLWDLISFLIPSLLLLVVFKAFDVRAFTRDG
```

Helix 10

```
HMADTLLLLLLYGWAIIPLMYLMNFFFLGAA
HMADTLLLLLLYGWAIIPLMYLMNFFFLGAA
HMADTLLLLLLYGWAIIPLMYLMNFFFLGAA
HMADTLLLLLLYGWAIIPLMYLMNFFFLGAA
HMADTLLLLLLYGWAIIPLMYLMNFFFLGAA
HMADTLLLLLLYGWAIIPLMYLMNFFFLGAA
HMADTLLLLLLYGWAIIPLMYLMNFFFLGAA
HMADTLLLLLLYGWAIIPLMYLMNFFFLGAA
```

Helix 11

```
TAYTRLTIFNILSGIATFLMVTIMRIPAVK
TAYTRLTIFNILSGIATFLMVTIMRIPAVK
TAYTRLTIFNILSGIATFLMVTIMRIPAVK
TAYTRLTIFNILSGIATFLMVTIMRIPAVK
TAYTRLTIFNILSGIATFLMVTIMRIPAVK
TAYTRLTIFNILSGIATFLMVTIMRIPAVK
TAYTRLTIFNILSGIATFLMVTIMRIPAVK
TAYTRLTIFNILSGIATFLMVTIMRIPAVK
```

Helix 11b

```
LEELSKTLDHVFLVLPNHCLGMAVSSFYENYETRRYCT



LEELSKTLDHVFLVLPNHCLGMAVSSFYENYETRRYCT

LEELSKTLDHVFLVLPNHCLGMAVSSFYENYETRRYCT
```

Helix 12

```
SAPGVGRFVASMAASGCAYLILLFLIETNLLQ
SAPGVGRFVASMAASGCAYLILLFLIETNLLQ
SAPGVGRFVASMAASGCAYLILLFLIETNLLQ
SAPGVGRFVASMAASGCAYLILLFLIETNLLQ
SAPGVGRFVASMAASGCAYLILLFLIETNLLQ
SAPGVGRFVASMAASGCAYLILLFLIETNLLQ
SAPGVGRFVASMAASGCAYLILLFLIETNLLQ
SAPGVGRFVASMAASGCAYLILLFLIETNLLQ
```

Figure S1. Prediction of TMD1 and TMD2 helix domains (in red) from sequences reported by different authors/software. For Bruder E. *et al*. 2007 see reference [36], for Park S.K. *et al*. 2010 see reference [37].

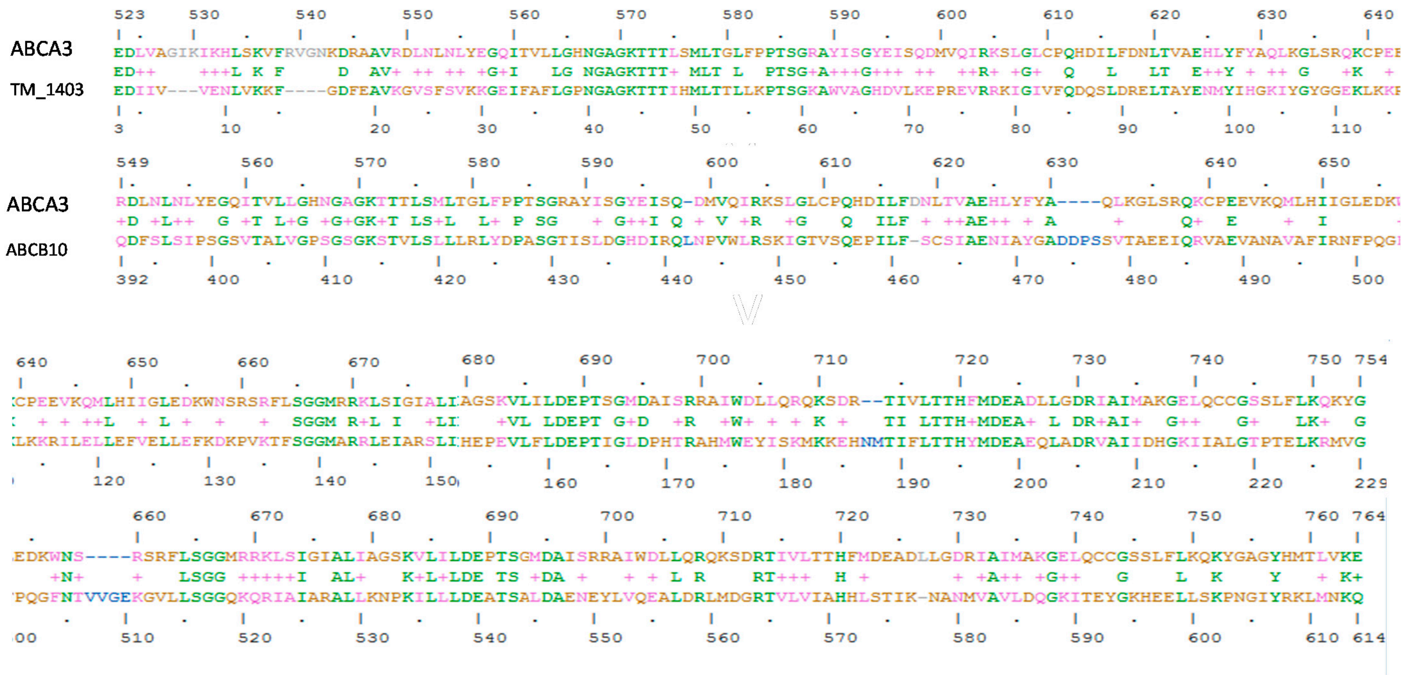

(**A**)

Figure S2. *Cont.*

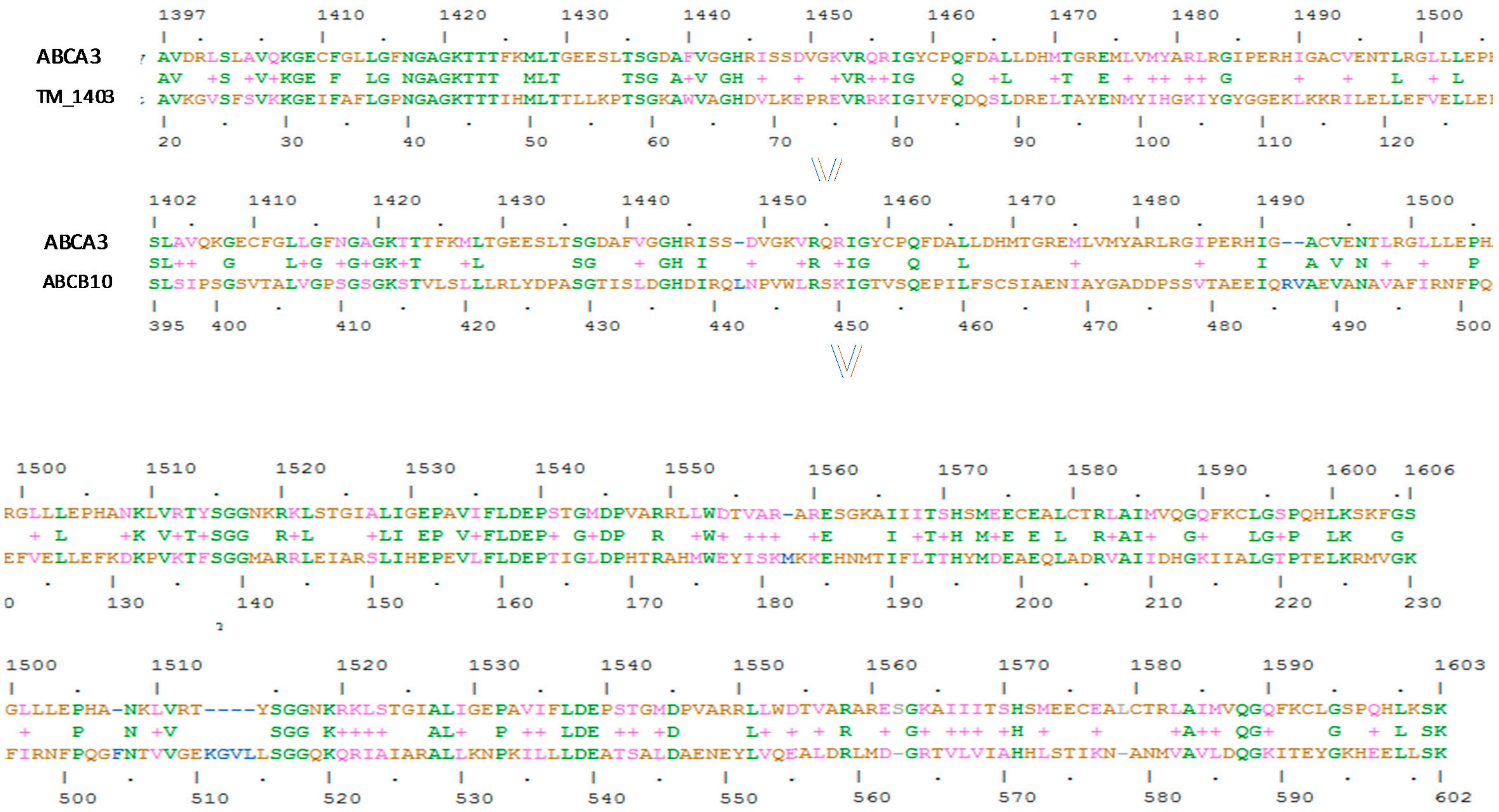

(B)

Figure S2. From the secondary structure comparative analyses on the ABCA3 protein we found that NBD1 (**A**) and NBD2 (**B**) respectively have 35% and 38% similarity with the correspondent NBDs of *Thermotoga maritime* (TM_1403), and a similarity of 28% and 23% respectively with NBDs of human ABCB10. In the alignment, amino acids are represented in green (when amino acids are the same), pink (when the amino acid lateral groups are similar), orange (when amino acid9.s are different), blue (when a deletion occurred) and grey (when an insertion occurred).
